# Supplementary material for: Urinary Multidrug-Resistant Klebsiella pneumoniae: Essential Oil Countermeasures in a One Health Case Report
Source: Microorganisms. 2025 Aug 1;13(8):1807. doi: 10.3390/microorganisms13081807 (PMC12388092; doi:10.3390/microorganisms13081807)
Supplement: Supplementary file 1 [file microorganisms-13-01807-s001.zip › microorganisms-3781224-supplementary.pdf]

Supplementary file

The supplementary file is a compact photographic atlas that documents every phenotypic test performed on the *Klebsiella pneumoniae* isolate discussed in the manuscript. It is arranged in the logical order in which the assays were run:

- **Colony morphology (4 images).** Four high-resolution photographs show the typical appearance of the strain on Mueller-Hinton, UriSelect, blood-agar and MacConkey plates, respectively, providing a quick visual check of pigmentation, haemolysis and lactose fermentation traits Supplementary material.
- **Single-antibiotic susceptibility (7 images).** Eleven Petri-dish photographs illustrate disk-diffusion results for 44 individual agents covering all major antimicrobial classes. Each image is captioned with the drug name, its coded abbreviation, the measured inhibition-zone diameter (when recorded) and the categorical interpretation (S or R).
- **Antibiotic-combination plate (1 image).** A dedicated dish (labelled "Petri dish 8") captures the simultaneous testing of five drugs, plus a triple-disk section that explores the combined effect of florfenicol, tulathromycin and streptomycin.
- **Essential-oil activity (2 images).**
    - *Petri dish 6* shows six pure essential oils (palmarosa, geranium, frankincense, laurel, tea-tree, citronella) with their respective inhibition zones.
    - *Petri dish 7* presents a second panel containing thyme oil, propolis tincture and the commercial phytotherapeutic blend **Biomicin Urinar (A20)** Supplementary material.
- **Mixed assay (1 image).** *Petri dish 9* places an antibiotic disk alongside essential-oil disks and a methylene-blue well, illustrating how these disparate compounds interact on the same lawn.
- **Commercial phytotherapeutic product (1 image).** *Petri dish 10* focuses exclusively on **Biomicin Forte (A3/Fares)**, a thyme- and clove-oil preparation that produced a 20 mm inhibition zone

Appearance of colonies on different culture media

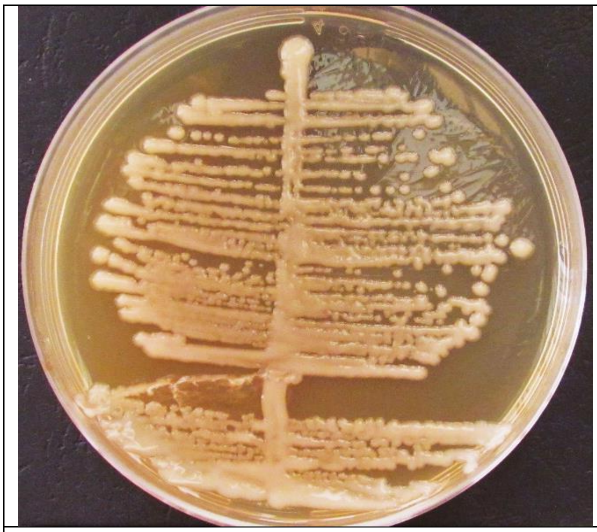	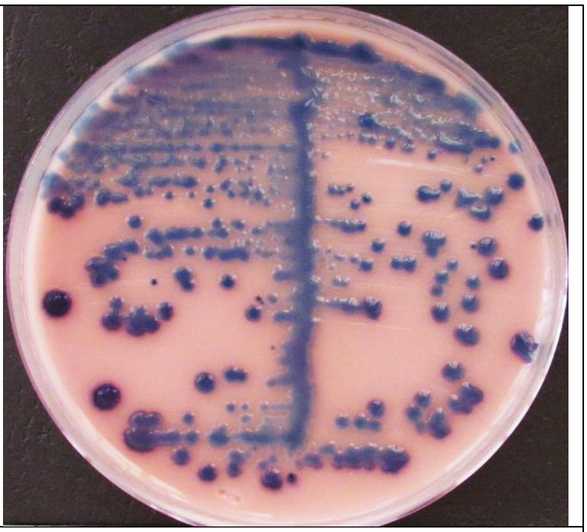
Fig. 1. Colonies on Mueller-Hinton medium	Fig. 2. Colonies on Uriselect medium
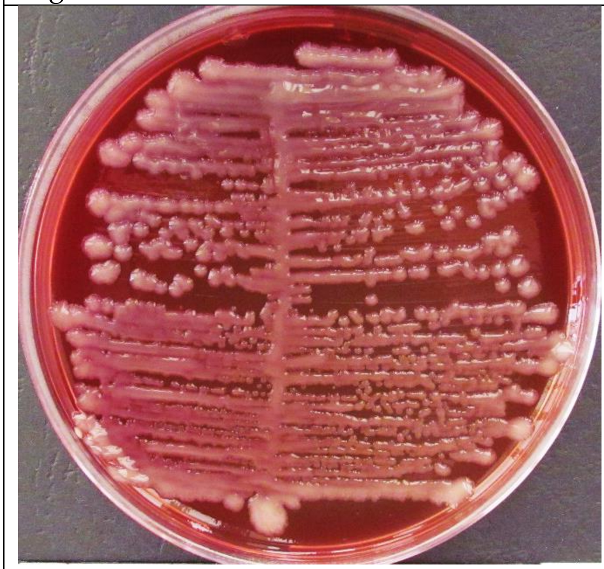	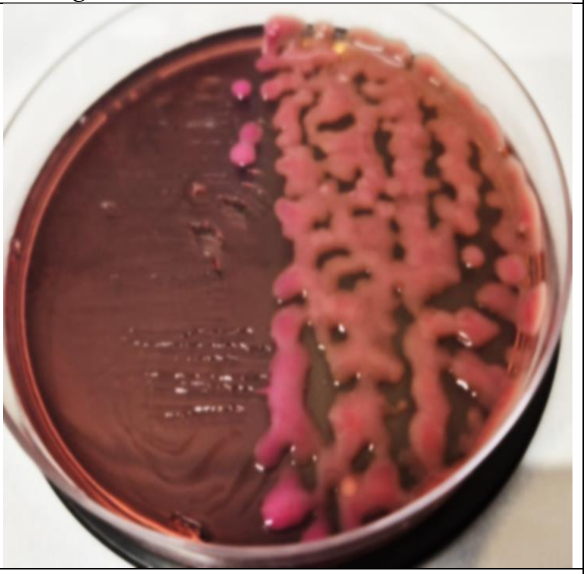
Fig. 3 Colonies on blood agar	Fig. 4 – Colonies on MacConkey agar

Aspect of antibiotic-susceptibility Petri dishes

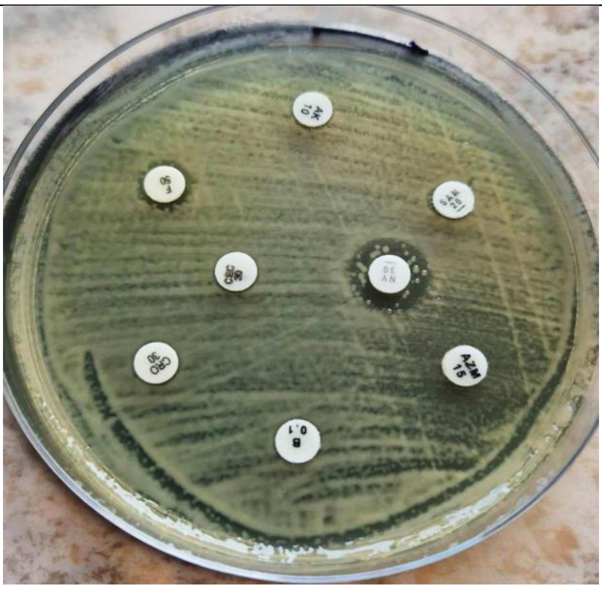	**Petri dish 1 (antibiotics)**<br>1. Amikacin (AK/) - R<br>2. Ampicillin with sulbactam(SAM) - R<br>3. Azithromycin (AZM) - R<br>4. Bacitracin (DTD) - R<br>5. Ceftriaxone (CRO) - R<br>6. Nitrofurantoin - R<br>7. Cefaclor (CEC) - R<br>8. Novobiocin (NV)* 11 mm
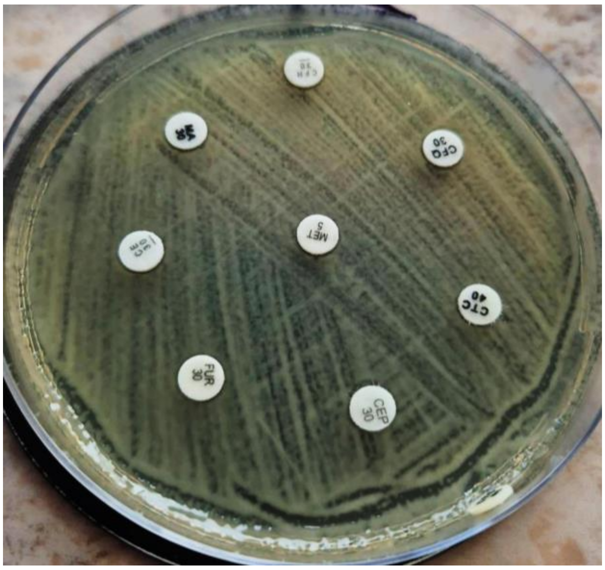	**Petri dish 2 (antibiotics)**<br>1. Cefadroxil (CFR) – R<br>2. Cefquinome (CFQ) – R<br>3. Cefotaxime with clavulanic acid (CTC) – R<br>4. Cefoperazone (CEP) – R<br>5. Ceftiofur (FUR) – R<br>6. Cephradine (CE) – R<br>7. Cefamandole (MA) – R<br>8. Metronidazole (MET) – R

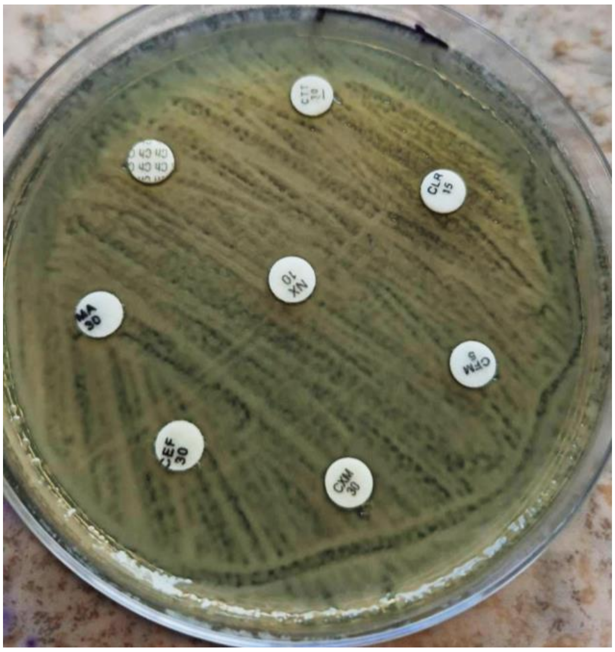

Petri dish 3 (antibiotics)

1. Cefotetan (CTT) – R
2. Clarithromycin (CLR) – R
3. Cefixime (CFM) – R
4. Cefuroxime (CXM) – R
5. Cefacetrile (CEF) – R
6. Cefamandole (MA) – R
7. Cephalothin (CH) – R
8. Norfloxacin (NX) – R

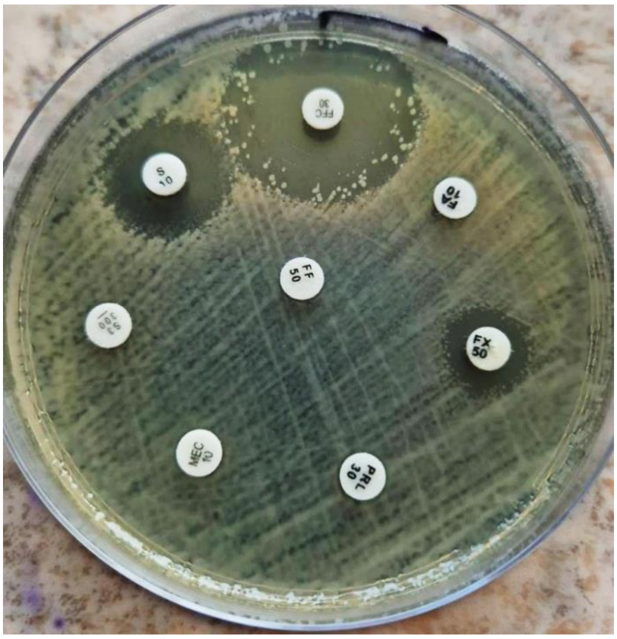

Petri dish 4 (antibiotics)

1. Florfenicol (FFC) 30 mm* – S
2. Fusidic acid (FA) – R
3. Furazolidone (FX) 11 mm – S
4. Piperacillin (PRL) – R
5. Mecillinam (MEC) – R
6. Compound sulphonamides (S3) – R
7. Streptomycin (S) 16 mm – S
8. Fosfomycin (FF) – R

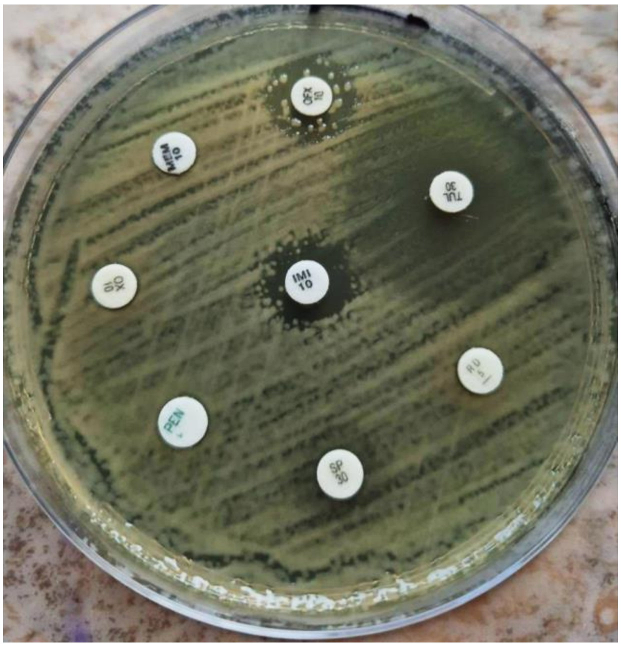

Petri dish 5 (antibiotics)

1. Ofloxacin (OFX) 14 mm* - S
2. Tulathromycin (TUL) 30 mm* - S
3. Rifampicin (RD) - R
4. Spiramycin (SP) - R
5. Penicillin (PEN) - R
6. Oxacillin (OX) - R
7. Meropenem (MEM) - R
8. Imipenem (IMI) 12 mm* - S

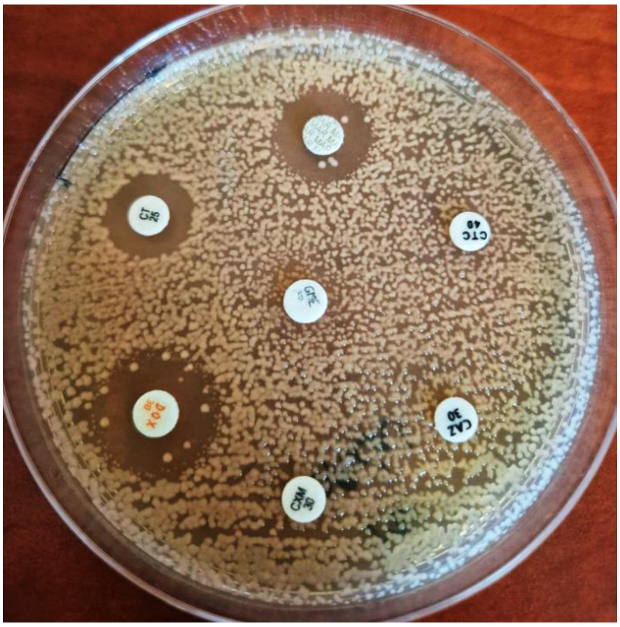

Petri dish 10 (antibiotics)

1. Marbofloxacin (MAR) 14 mm* - S
2. Cefotaxime + clavulanic acid (CTC) - R
3. Ceftazidime (CAZ) - R
4. Cefuroxime (CXM) - R
5. Doxycycline (DOX) 18 mm* - S
6. Colistin (CT) 12 mm* - S
7. Gentamicin (GME) - R

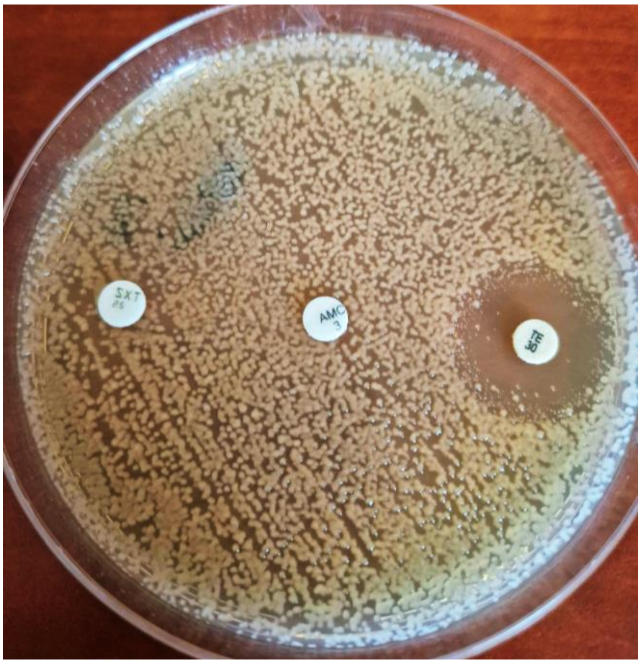

Petri dish 11 (antibiotics)

1. Trimethoprim + Sulfamethoxazole (SXT) - R
2. Amoxicillin + Clavulanic acid (AMC) - R
3. Tetracycline (TET) 20 mm* - S

Images of plates with antibiotic combinations

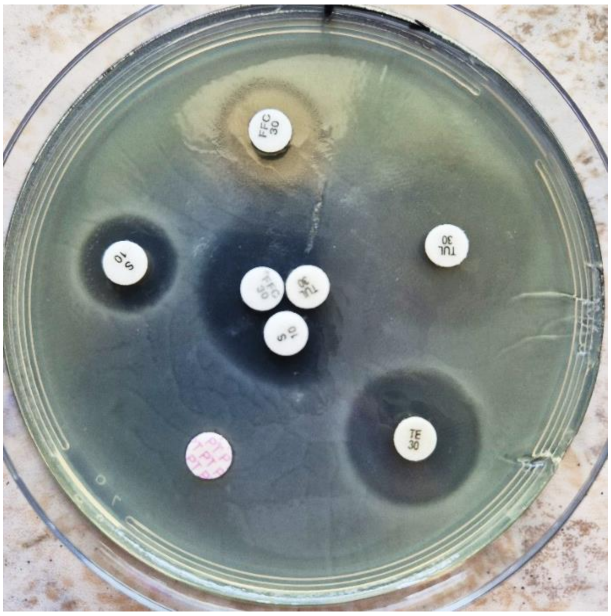

Petri dish 8 (antibiotic combinations)

1. Florfenicol (FF) 11.7 mm - S
2. Tulathromycin (TUL) 15.30 mm - S
3. Tetracycline (TE) 12.4 mm - S
4. Pristinamycin (PT) - R
5. Streptomycin (S) 10.76 mm – S

Combination of 3 antibiotics

6. Florfenicol (FF)
7. Tulathromycin (TUL)
8. Streptomycin (S)

Images of Petri dishes with essential oils

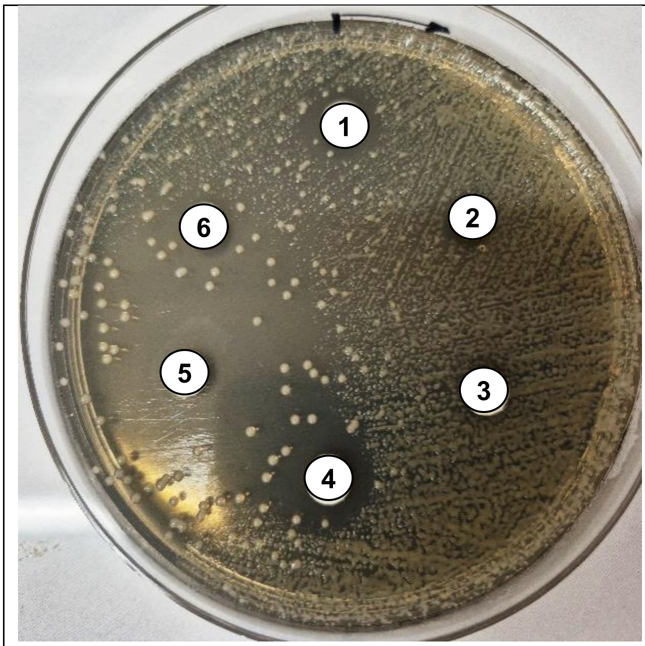

Petri dish 6 (essential oils)

1. Palmarosa (*Cymbopogon martini*)(11 mm) - S
2. Geranium (*Geranium intense*) - R
3. Frankincense (*Boswellia* sp.) - R
4. Laurel (*Laurus nobilis*) (12 mm) - S
5. Tea tree (*Melaleluca alternifolia*) (20 mm) - S
6. Citronella (*Cymbopogon nardus*) - R

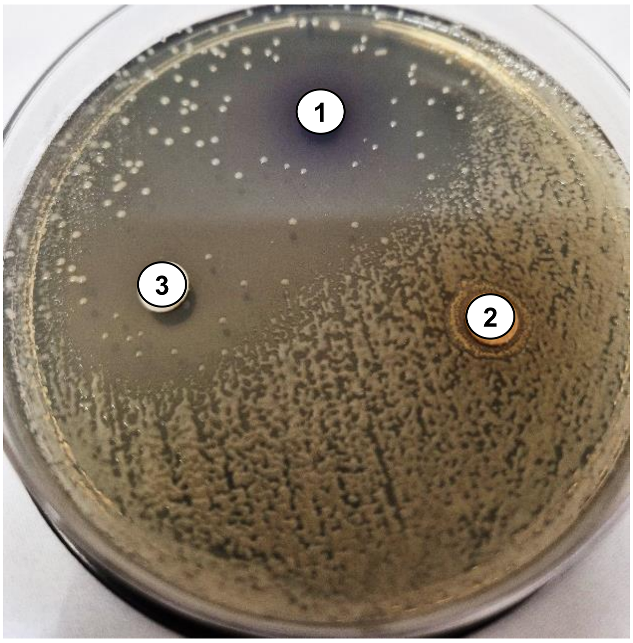 	**Petri dish 7 (essential oil + natural preparations)**<br><br>1. Thyme (*Summer savory/Satureja hortensis)* (12 mm)* - S<br>2. Propolis tincture (natural mixture of resins and beeswax) - R<br>3. Biomicin Urinar (A20) 22 mm* - S<br><br>Formulation containing:<br>• Oregano essential oil (*Origani aetheroleum, Origanum vulgare*)<br>• Cinnamon essential oil (*Cinnamomum verum*)<br>• Sage essential oil (*Salvia officinalis*)<br>• Cultivated thyme essential oil (*Thymi aetheroleum, Thymus vulgaris*)
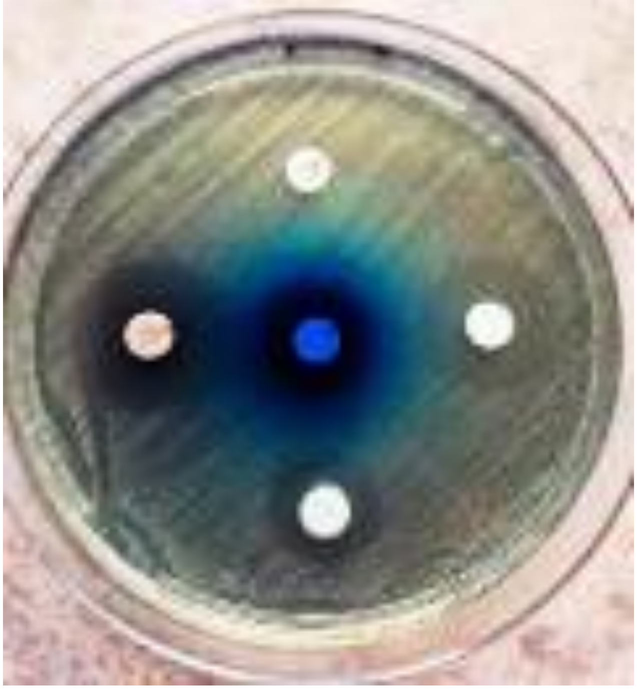	**Petri dish 9 (antibiotic + essential oils + methylene blue)**<br><br>1. Amoxicillin + clavulanic acid - R<br>2. Tea tree (*Melaleuca alternifolia)* (17.99 mm) – S<br>Biomicin Urinar (A20) 13.96 mm - S<br>Formula containing: oregano essential oil (*Origani aetheroleum*), cinnamon essential oil (*Cinnamomum verum*), sage essential oil (*Salvia officinalis*), and others<br>3. Thyme (*Summer savory/Satureja hortensis)* (18.05 mm) - S<br>*Summer savory / Satureja hortensis*<br>4. Methylene blue (12.33 mm, 1 % solution) - S

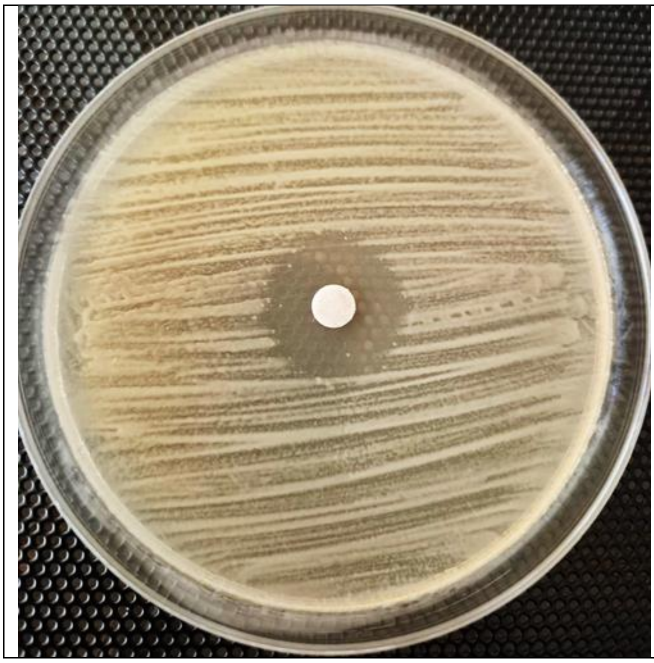	**Petri dish 10 - Biomicin Forte (A3/Fares) – Thyme essential oil (thyme & clove)**<br><br>Biomicin Forte (A3/Fares) – 20 mm inhibition zone - S<br>Formulated with:<br>• Thyme essential oil (*Thymi aetheroleum*)<br>• Clove essential oil (*Caryophyllorum aetheroleum*)
